# Newborn Screening for Fabry Disease in Northeastern Italy: Results of Five Years of Experience

**DOI:** 10.3390/biom11070951

**Published:** 2021-06-27

**Authors:** Vincenza Gragnaniello, Alessandro P Burlina, Giulia Polo, Antonella Giuliani, Leonardo Salviati, Giovanni Duro, Chiara Cazzorla, Laura Rubert, Evelina Maines, Dominique P Germain, Alberto B Burlina

**Affiliations:** 1 Division of Inherited Metabolic Diseases, Department of Diagnostic Services, University Hospital, 35129 Padua, Italy; vincenza.gragnaniello@aopd.veneto.it (V.G.); giulia.polo@aopd.veneto.it (G.P.); antonella.giuliani@aopd.veneto.it (A.G.); chiara.cazzorla@aopd.veneto.it (C.C.); laura.rubert@aopd.veneto.it (L.R.); 2 Neurology Unit, St Bassiano Hospital, 36061 Bassano del Grappa, Italy; alessandro.burlina@aulss7.veneto.it; 3 Clinical Genetics Unit, Department of Diagnostic Services, University Hospital, 35128 Padua, Italy; leonardo.salviati@unipd.it; 4 Institute for Biomedical Research and Innovation, National Research Council of Italy (IRIB CNR), 90146 Palermo, Italy; giovanni.duro@ibim.cnr.it; 5 Division of Pediatrics, S. Chiara General Hospital, 38122 Trento, Italy; evelina.maines@apss.tn.it; 6 Division of Medical Genetics, University of Versailles and APHP Paris Saclay University, 92380 Garches, France; dominique.germain@uvsq.fr

**Keywords:** Fabry disease, newborn screening, variant interpretation, second tier test, tandem mass spectrometry, lyso-Gb_3_, dried blood spot, α-galactosidase A, *GLA* gene, globotriaosylsphingosine

## Abstract

Fabry disease (FD) is a progressive multisystemic lysosomal storage disease. Early diagnosis by newborn screening (NBS) may allow for timely treatment, thus preventing future irreversible organ damage. We present the results of 5.5 years of NBS for FD by α-galactosidase A activity and globotriaosylsphingosine (lyso-Gb_3_) assays in dried blood spot through a multiplexed MS/MS assay. Furthermore, we report our experience with long-term follow-up of positive subjects. We screened more than 170,000 newborns and 22 males were confirmed to have a *GLA* gene variant, with an incidence of 1:7879 newborns. All patients were diagnosed with a variant previously associated with the later-onset phenotype of FD or carried an unclassified variant (four patients) or the likely benign p.Ala143Thr variant. All were asymptomatic at the last visit. Although lyso-Gb_3_ is not considered a reliable second tier test for newborn screening, it can simplify the screening algorithm when its levels are elevated at birth. After birth, plasma lyso-Gb_3_ is a useful marker for non-invasive monitoring of all positive patients. Our study is the largest reported to date in Europe, and presents data from long-term NBS for FD that reveals the current incidence of FD in northeastern Italy. Our follow-up data describe the early disease course and the trend of plasma lyso-Gb_3_ during early childhood.

## 1. Introduction

Fabry disease (FD, OMIM 301500) is an X-linked lysosomal disorder (LSD) caused by a deficiency of α-galactosidase A (α-GAL A) activity that results in the progressive accumulation of globotriaosylceramide (Gb_3_) and related glycosphingolipids, particularly in cellular lysosomes and body fluids [1,2]. The clinical phenotype includes a broad spectrum of clinical severity ranging from classic to later-onset FD. Male patients with the classic FD phenotype may present in childhood with acroparesthesias, angiokeratomas, corneal opacities, gastrointestinal symptoms, neuropathic pain, and hypohidrosis, followed in adulthood by renal failure, cardiac and cerebrovascular disease, and premature death [3,4]. Men with the later-onset phenotype of FD invariably present with cardiovascular involvement (hypertrophic cardiomyopathy with arrhythmias and conduction abnormalities), with very rare occurrences of renal (albuminuria, proteinuria) and cerebrovascular involvement [5]. In heterozygous females, clinical manifestations vary from asymptomatic to the classic severe phenotype, largely depending on X-chromosome inactivation [6]. Diagnosis in affected males can be achieved by the α-GAL A enzyme activity assay on several sample matrix types (dried blood spot DBS, peripheral white blood cells or plasma) and/or by molecular analysis [7,8]. α-GAL A enzyme activity levels may be normal in heterozygous females due to X-chromosome inactivation in blood cells, so that gene testing is necessary [1]. Analysis of biomarkers (e.g., Gb_3_ and its deacylated form, lyso-Gb_3_) in plasma, urine, and DBS are useful for both diagnosis and follow-up [9,10,11]. Enzyme replacement therapy (ERT) with agalsidase alfa or agalsidase beta [12,13] and chaperone therapy (for “amenable” *GLA* genetic variants) are available; expert panels have provided recommendations for clinical management [14,15]. Early intervention plays an important role in preventing irreversible damage due to disease progression [16].

Recently, pilot newborn screening (NBS) programs for FD, based on enzyme activity assay in DBS have been implemented worldwide using several analytical techniques. Initially, a fluorescent method with 4-methylumbelliferyl-D-galactopyranoside as a substrate was used [17]. Later, multiplexed techniques were developed using digital microfluidic fluorometric and MS/MS technology [18,19,20,21]. Several NBS programs in the USA (Washington State, Missouri, Illinois) [22,23,24], Europe (Hungary, Austria, Spain, Italy) [25,26,27,28], and East Asia (Taiwan, Japan) [29,30] showed that FD is surprisingly more prevalent than previously estimated (1:40,000) [31], especially the later-onset form, which may represent an important unrecognized genetic disease, although the caveats and difficulties of variant interpretation should be paid a lot of consideration [32]. A summary of available data on the known NBS programs for FD worldwide is presented in Table 1.

## 2. Materials and Methods

### 2.1. Study Population

From September 2015 until March 2021, the DBS from 173,342 newborns were collected consecutively by the Regional North East Italy expanded neonatal screening program. The DBSs were assayed for the enzymes deficient in FD and three other lysosomal diseases (LSD-Pompe disease, Gaucher disease, mucopolysaccharidosis type I). Written informed consent was obtained from a parent. Proof of informed consent is available upon request. According to the NBS protocol, samples were collected between 36 and 48 h of life on the same card used for the other NBS tests; a second sample was required for premature newborns (<34 gestational weeks and/or weight <2000 g) and for sick newborns (those receiving transfusion or parenteral nutrition). DBS were analyzed on the day they were received, and the DBS cards were stored at −20 °C in plastic bags for at least five years after analysis. All analyses were performed in compliance with institutional review board guidelines.

### 2.2. Methods

α-GAL A enzyme activity was determined simultaneously with acid α-glucosidase (deficient in Pompe disease), β-glucosidase (deficient in Gaucher disease), and α-L-iduronidase (deficient in mucopolysaccharidosis type I) using a multiplex MS/MS assay (PerkinElmer, Turku, Finland). Assay results were obtained after overnight incubation and enzyme activity was expressed as micromoles of substrate hydrolyzed per hour of incubation per liter of blood (µmol/L/h). Samples with low activities for several enzymes were repeated due to a suspected preanalytical error. For IDUA activity, an initial cut-off of 3.76 µmol/L/h was established using 0.2 multiples of the median (MOM); this was reset to 2.3 µmol/L/h after the first 9 months of screening [28]. Samples with α-GAL A activity below the cut-off were retested in duplicate. If the mean of the enzyme activity values was confirmed to be low, a second DBS was requested and assayed using the same cut-off. Newborns with abnormal enzyme results on the second round were referred to the Division of Inherited Metabolic Disease, Padua University Hospital, for confirmatory testing and clinical follow-up. DBS with low α-GAL A activity were also tested for lyso-Gb_3_ using the LC-MS/MS method with a cut-off value of 1.3 nmol/L. This cut-off was established from the analysis of 253 anonymous healthy adult blood donors (*n* = 133) and pediatric patients (*n* = 120), and the 97.5 th percentile was considered [10].

Confirmatory testing included α-GAL A enzyme activity in peripheral lymphocytes, plasma lyso-Gb_3_ measurement, and molecular analysis. Moreover, the mothers of confirmed newborns underwent *GLA* molecular testing. α-GAL A enzyme activity in lymphocytes was measured initially using a fluorometric method and more recently using a MS/MS assay. lyso-Gb_3_ was measured in plasma samples using LC-MS/MS technology, as described in [10]. The *GLA* gene was sequenced using Next Generation Sequencing technology on genomic DNA isolated from peripheral leukocytes. Variants were classified according to published clinical reports and public databases including the International Fabry Disease Genotype-Phenotype Database and The Fabry Working Group Genotype Phenotype Database [49,50,51].

Patients were monitored every 12 months, with clinical evaluation: angiokeratomas, hypohidrosis, gastrointestinal symptoms, limb pain, kidney (eGFR according to Schwartz formula, microalbuminuria, proteinuria), and cardiac (electrocardiogram) assessments. Plasma lyso-Gb_3_ was monitored as a specific marker of the disease. 

## 3. Results

All results of the NBS program for FD are summarized in Table 2. Of the 173,342 newborns screened (89,485 males and 83,857 females) from September 2015 to March 2021, 53 (44 males and 9 females) had a low α-GAL A enzyme activity in the first DBS and were recalled for a second spot (recall rate 0.03%). Lowα-GAL A activity was confirmed in 23 newborns (22 males and one female) at the second DBS and referred to our outpatient Clinical Unit for confirmatory testing. All 22 males from 20 families were confirmed to have a decreased α-GAL A enzyme activity in lymphocytes and a genetic variant in the *GLA* gene, with an incidence of one in 4068 males. The only female newborn with a measured low DBS α-GAL A activity was negative at molecular testing. Regarding the ethnicity of the 22 positive neonates, four were of African origin, two were of Asian origin, and the others were Caucasian. Patients #13 and #20 and patients #16 and #21 were brothers. None of the patients had a family history of a previous FD diagnosis or manifestations strongly suggestive of FD (e.g., angiokeratomas, cornea verticillata, end-stage renal failure). The clinical, biochemical, and molecular features of the patients are presented in Table 3. 

### 3.1. Enzyme Activity

The α-GAL A enzyme activities in DBS ranged from 0.63 to 3.45 µmol/L/h (*n* = 22, mean 1.54 µmol/L/h, SD 0.88). After confirmatory testing, we found that the α-GAL A enzyme activities of males with known pathogenic *GLA* variants ranged from 0.63 to 1.88 µmol/L/h (*n* = 13, mean 1.07 µmol/L/h, SD 0.43), whilst in males carrying unclassified *GLA* variants or the p.Ala143Thr variant, it ranged from 0.87 to 3.45 µmol/L/h (*n* = 8, mean 2.21 µmol/L/h, SD 1.02). This difference was statistically significant (*p* = 0.003, according to the Student’s *t*-test).

### 3.2. Genetic Testing

Molecular analysis identified 13 newborns (including two brothers) carrying known pathogenic variants (11 had a missense variant, two had an intronic splicing variant) associated with the later-onset form of FD, four carrying previously unclassified GLA variants, four with the p.Ala143Thr variant, and one carrying a haplotype considered to be benign (patient #5). Among our patients, the most common variant was p.Ala143Thr (four patients), followed by p.Asn215Ser (three patients). This latter is a known pathogenic variant found in association with the later-onset cardiac form of FD [5,52], while the p.Ala143Thr, which had initially been classified as pathologic now has its pathogenicity of conflicting interpretation with a number of reports in favor of a likely benign variant [50,53]. Patients #7 and #9, unrelated newborns of Asiatic origin, carried the splicing variant IVS4 + 919G>A, which is common in the Taiwanese population and is associated with a later-onset cardiac phenotype [42]. Moreover, we found two unrelated patients from northern Africa carrying the p.Arg363His variant. Of note, we frequently found the benign complex allele −10 C > T + IVS2-77_81del5 + IVS4-16A > G + IVS6-22C > T polymorphism, in association with other variants. It was identified in the absence of other *GLA* variation in patient #5, who had near normal enzyme activity. All mothers carried the same *GLA* variant of their respective offspring.

### 3.3. DBS Lyso-Gb_3_

We started DBS lyso-Gb_3_ testing in 2016, and tested 17 out of the 22 positive newborns. The values ranged from 0.22 to 2.7 nmol/L (mean 1.04 nmol/L, SD 0.65). Levels were abnormal in five newborns (patients #8, #10, #12, #18, and #19), among the 12 tested patients carrying a known later-onset variant (mean 1.8 nmol/L, SD 0.70). No patient carrying unclassified or likely benign variants had abnormal levels of DBS lyso-Gb_3_ at birth (*n* = 5, mean 0.72 nmol/L, SD 0.35). This difference was not statistically significant (*p* = 0.188, according to the Student’s t-test), but this is likely due to the small number of samples. However, an inverse linear correlation was found between DBS α-GAL A activity and DBS lyso-Gb_3_ at birth (*r* = −0.28).

### 3.4. Plasma Lyso-Gb_3_

At the first visit, we evaluated plasma lyso-Gb_3_ in all neonates (except in case #20 due to technical problems) and it ranged from 0.12 to 2.98 nmol/L (mean 0.66 nmol/L, SD 0.63). It was above the cut-off in 11/21 patients, nine of which had a known pathogenic variant. lyso-Gb_3_ was only slightly increased in patient #1 (p.Ala143Thr variant) and in patient #16 (p.Leu286Val, unclassified variant), whilst the highest values were observed in patients with the known pathogenic variants p.Asn215Ser, p.Arg363His, and, interestingly, in patient #10 (p.Arg356Gly), which is listed as a likely later-onset form in the International Fabry Disease Genotype-Phenotype Database [49]. The mean of values for patients with known pathogenic variants was 0.90 ± 0.75 nmol/L, while for patients carrying unclassified variants or the likely benign p.Ala143Thr variant, it was 0.36 ± 0.13 nmol/L. This difference was not statistically significant (*p* = 0.061, according to Student’s t-test). However, an inverse linear correlation, stronger than the one between DBS α-GAL A activity and DBS lyso-Gb_3_, was found between DBS α-GAL A activity and plasma lyso-Gb_3_ at first visit (*r* = −0.41).

### 3.5. Follow-Up

**Figure 1:**
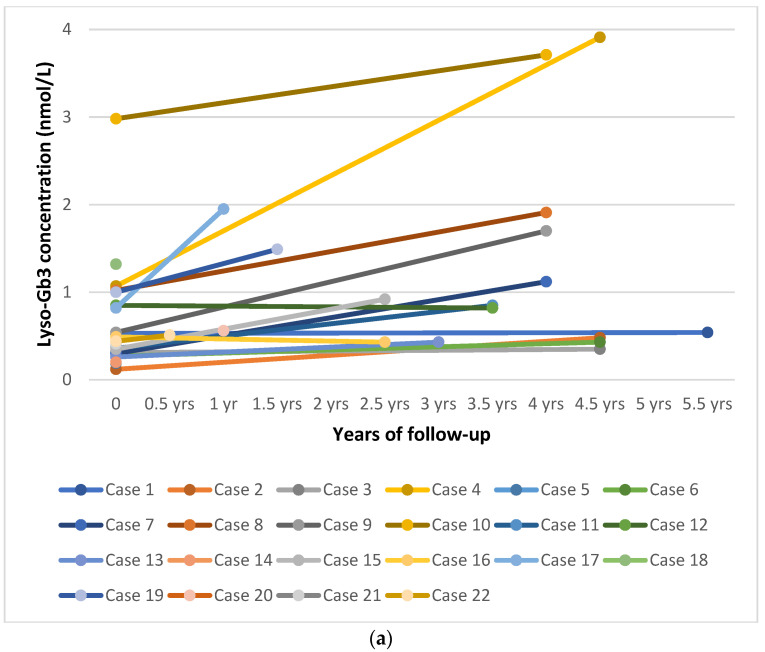

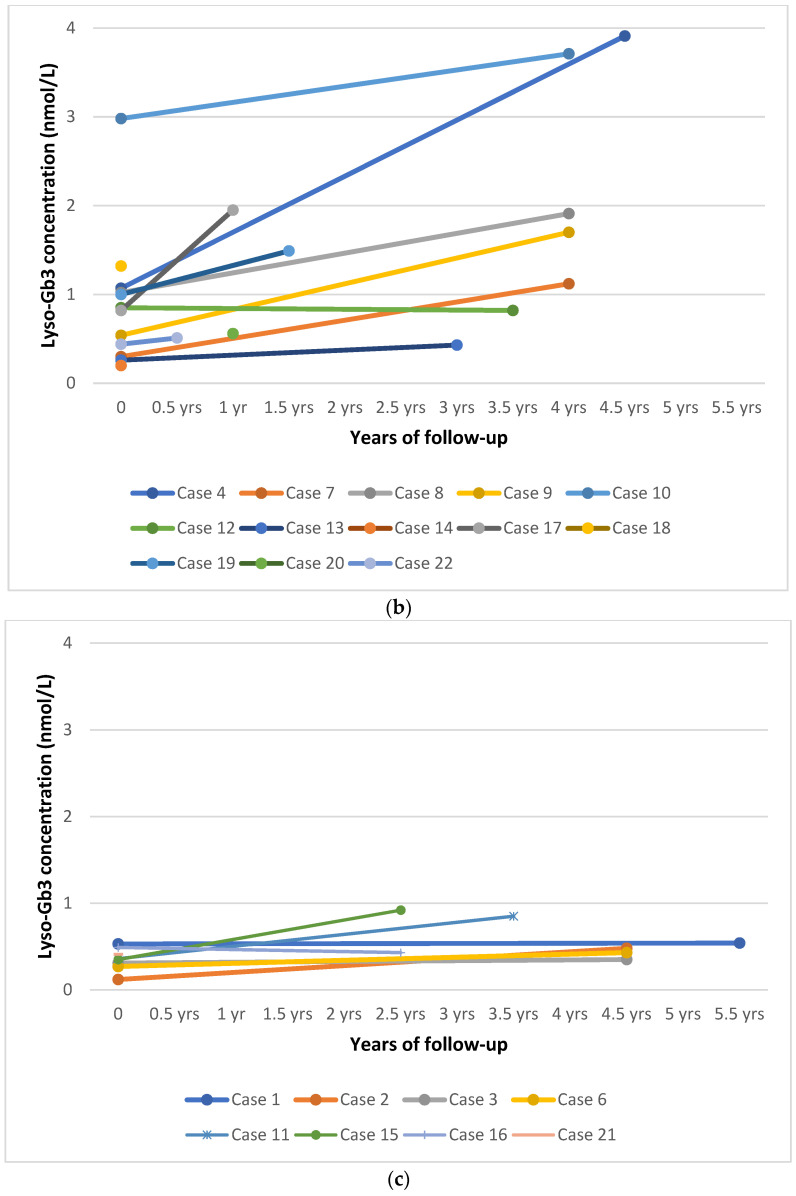
(**a**) Trend in plasma lyso-Gb_3_ levels over time in all subjects positive for Fabry disease. (**b**) Plasma lyso-Gb_3_ levels over time in patients carrying later-onset variants. (**c**) Plasma lyso-Gb_3_ levels over time in subjects carrying benign and unclassified variants (including p.Ala143Thr). Abbreviation: yrs: years.

None of the patients showed clinical or biochemical abnormalities at the first visit. All patients participated in regular follow-up except patients #5 and #14, who were lost to follow-up because parents refused additional medical examinations. A summary of clinical and biochemical features at the first visit and during follow-up is reported in Table 3 and Table S1. All patients were asymptomatic at the latest follow-up visit at the age indicated in Table 3. None of the patients were receiving specific treatment for FD. The mean plasma lyso-Gb_3_ level was 1.19 nmol/L SD 1.06 (range 0.35–3.91 nmol/L), with slightly elevated values in 17 patients at the latest visit (85%). Interestingly, plasma lyso-Gb_3_ levels increased in most children (mean annual increase 0.21 ± 0.29 nmol/L; ranges between –0.024 nmol/L in case #16 and 1.13 nmol/L in case #17; Figure 1). Higher values were found in patients carrying a known pathogenic variant (*n* = 11, range 0.43–3.91 nmol/L, mean 1.65, SD 1.20, mean annually increase 0.32 ± 0.33 nmol/L) than in individuals carrying unclassified variants or the variant p.Ala143Thr (*n* = 7, range 0.4–0.92 nmol/L, mean 0.55, SD 0.21, mean annual increase 0.06 ± 0.09 nmol/L). This difference was statistically significant (*p* = 0.033, Student’s t-test). The p.Asn215Ser variant appeared to be associated with greater increase. Of note, all three patients with this variant had a value of 1 nmol/L at birth, with values increasing with age. The older patient had a value of 3.91 nmol/L at the age of 4.5 years.

## 4. Discussion

Here, we report our results from more than five years of NBS for FD in northeast Italy, based on the determination of α-GAL A enzyme activity in DBS using a multiplex MS/MS assay.

### 4.1. Epidemiology

In the last 5.5 years, we screened 173,342 newborns (89,485 males and 83,857 females) for FD, which is the largest study reported to date in Europe. *GLA* variants were identified in 22 males, 13 of which were known pathogenic variants previously reported in association with a later-onset phenotype. One was a benign haplotype, four were unclassified variants, and four carried the p.Ala143Thr variant whose pathogenicity is debated. The four patients with the p.Ala143Thr variant did not appear to have classic FD, based on the clinical picture and biomarkers. Thus, the overall incidence of α-Gal A deficiency was of 1 in 7879 (1 in 4068 males), while the frequency of pathogenic variants was 1 in 6883 males. This incidence was similar to those detected in our previous pilot study, conducted from September 2015 to January 2017 on about 40,000 births (incidence 1:8,822 newborns) [28]. Moreover, this incidence was about six times higher than originally estimated from the clinical data (1:40,000) [31] but similar to previous reports from NBS programs. This is likely due to the recognition of previously undiagnosed later-onset forms of FD. Indeed, our incidence is comparable to those reported by a previous Italian study, conducted in north Italy from July 2003 to June 2005, using a fluorometric enzyme assay on 37,104 consecutive males. Twelve infants with *GLA* variants were found (incidence of 1:3100 males) and only one had a mutation known to cause the classic phenotype [33]. The incidence found in other studies is presented in Table 1. However, it is difficult to make comparisons among studies because there are differences in screening techniques, numbers of newborns, geographical/ethnical variation, and changes in the classification of variants over time as knowledge accumulates. This last point makes the comparison among studies particularly difficult, because some previously pathogenic variants have been reclassified over the years, based on associated clinical and biochemical features and their high incidence in the population (e.g., p.Arg118Cys, p.Asp313Tyr, and the debated variant p.Ala143Thr). Moreover, regarding some unclassified variants, it is difficult to predict their pathogenicity because FD may occur later in life. For example, a Spanish study reported a very high incidence of disease (1:394 births), but the number of screened newborns was low (*n* = 14,600). Moreover, only one patient had a known pathogenic variant, while 25/37 carried benign variants [27]. Conversely, a program conducted in Illinois on a newborn population similar in size to our study (nearly 220,000) and using similar methodology (MS/MS assay) reported an incidence of 1:6868 births [24], which is comparable to our result. However, different genetic backgrounds can also explain differences in incidence between countries. In Taiwan, screening of nearly one million newborns revealed an FD incidence of 1:2078, because of the high incidence and founder effect of the later-onset *GLA* splicing variant (IVS4 + 919G > A) (82% of patients) [35,42]. All studies including our NBS program show that even when non-pathogenic variants are discarded, FD is much more frequent than previously thought based on clinical estimates; it is the most frequent LSD screened. These findings, associated with available diagnostic methods and treatments, support the importance of NBS.

### 4.2. Interpretation of Genetic Variants

The lack of knowledge about the long-term course of the disease, especially for later-onset forms, complicates the establishment of clear correlations between genotype and phenotype in FD. Affected hemizygotes with the classic disease manifestations and no detectable α-GAL activity are associated with a variety of *GLA* variants including large and small gene rearrangements, splicing defects, and missense or nonsense variants. In contrast, most mildly affected atypical hemizygotes usually bears missense variants that express residual α-GAL A activity. However, most Fabry patients have private variants and attempts to predict the clinical phenotype based on the type or location of a variant may prove difficult. Moreover, the influence of modifier genes or other genetic factors on phenotype severity may be confounders since individuals with the same *GLA* variant may occasionally have variable phenotypes including within the same family. Thus, the clinical severity of private missense variants detected in Fabry families with few, or only young patients, is difficult to predict and requires more extensive clinical information from unrelated patients with the same FD genotype [50]. Furthermore, the burden of common risk factors (e.g., hypertension, high levels of cholesterol, diabetes) and the presence of concomitant diseases can be responsible for adjunctive signs and symptoms during aging [4,54]. 

Among the 12 different variants that we found (seven known pathogenic variants, four unclassified variants, and the debated p.Ala143Thr variant), there were 11 missense variants and one intronic splicing variant. The most frequent variant (p.Ala143Thr) was found in four patients (Pts. 1, 2, 3 and 11) and is more frequent in the Caucasian population. In a previous Italian study, three of six positive males carried this variant [33], and a similar frequency was found in Austria where it was found in three of six positive males [26]. Moreover, more recently, a California NBS study also reported a high frequency of the p.Ala143Thr variant among positive newborns (22/50) [39]. This variant, previously associated with both classic [55] and later-onset phenotype [56], was subsequently considered benign [53,57]. It is relatively frequent in the general population and a previous study in COS cells demonstrated a high residual enzyme activity of 36% [33]. Moreover, reported individuals with this variant showed unspecific symptoms, but no increase of plasma Gb_3_ and lyso-Gb_3_ [58] and no storage in tissue biopsies [53,57]. However, the significance of this variant is still controversial. A Fabry disease genotype-phenotype working group recently analyzed unclassified GLA variants in the Fabry registry through a five-stage iterative system based on expert clinical assessment, published literature, and clinical evidence of pathogenicity, but the expert panel did not reach a definitive conclusion, and classified it as a variant of uncertain significance [50]. Our patients carrying this variant had high residual enzyme activity (about 29% of mean normal activity). Of note, three of our patients (pts. 1, 2, and 3) were identified during the first phase of our screening program, before adjustment of the cut-off for the α-GAL A activity. The fourth patient (patient #11), who had lower α-GAL A activity (2.05 µmol/L/h), also carried an intronic unclassified variant (IVS4-61_60delGT). It is possible that the association of these two variants could produce a further reduction in enzyme activity. All our patients carrying the variant p.Ala143Thr maintained normal or very slightly increased levels of plasma lyso-Gb_3_ during follow-up. Plasma lyso-Gb_3_ was higher in the patient with p.Ala143Thr plus the intronic unclassified variant (pt. 11; mean lyso-Gb_3_ 0.55 nmol/L, SD 0.21 at age 4.5 years); however, it was lower than the lyso-Gb_3_ levels in patients carrying known pathogenic variants (mean 1.62 nmol/L, SD 1.20). In our opinion, the α-GAL A activity and the lyso-Gb_3_ levels detected in the four babies with the genetic variant p.Ala143Thr are in favor of a likely benign classification of this variant. The high frequency of this variant in the gnomAD database (5.06 × 10^−4^) is also in favor of a benign variant relatively frequent in the European population (found in 88 of 92,769 European alleles) [59].

Among the pathogenic variants, we found a high frequency of the mutation p.Asn215Ser (patients #4, #17, and #18), associated with very low enzyme activity in DBS (0.64, 0.63, and 1.4 µmol/L/h, respectively). This variant is reported in patients with predominant cardiac involvement [5,52]. Other variants seem to be correlated with ethnic origin. Two unrelated Eastern Asiatic infants (from South China) carried the IVS4 + 919G>A variant. Interestingly, the DBS α-GAL A activity values of patients carrying a known pathogenic variant were significantly lower than the values of the patients with an unclassified variant or the likely benign p.Ala143Thr variant (*p* = 0.003).

### 4.3. Clinical Follow-Up

All patients were monitored, except for two who did not want to come back to the hospital periodically. Because all our patients carried variants associated with the later-onset form or unclassified variants, we decided to follow them up every 12 months. The follow-up included clinical, instrumental, and biochemical assessments: the search for angiokeratomas, limb pain, hypohidrosis, gastrointestinal symptoms, renal, and cardiac evaluations (eGFR according to Schwartz formula, microalbuminuria, proteinuria, cardiologic visit with ECG) (Table S1). We detected four newborns with unclassified variants and followed their clinical outcomes. Patient #6, carrying the p.Thr246Ile variant, had a high DBS α-GAL A activity (3.45 µmol/L/h). Currently at age 4.5 years, he has no signs or symptoms of disease and plasma lyso-Gb_3_ values correspond to the upper limit of the normal range (0.43 nmol/L), questioning pathogenicity of this variant. Patient #15 carried two variants (p.Gly116Ala) and the likely benign p.Ser126Gly and several benign intronic variants. He has no signs of disease at age 2.5 years, but plasma lyso-Gb3 level is slightly elevated (0.92 nmol/L), so that further follow-up visits are requested. Finally, two brothers (patients #16 and #21) carried the p.Leu286Val variant. The first brother is asymptomatic at two years of age, and has a plasma lyso-Gb_3_ value that corresponds to the upper limit of normal range (0.43 nmol/L), while the second brother is a newborn. We will continue to monitor them. Because FD can undergo silent progression without evident clinical manifestations, especially with later-onset forms, we believe that long-term follow-up is important. Indeed, Hsu et al. demonstrated that cardiac damage could progress in silence, even when it becomes severe and irreversible (e.g., cardiac fibrosis) [42], whilst Öqvist et al. emphasized the importance of early diagnosis and NBS in FD-related nephropathy [60]. We also carefully assessed limb pain, which can manifest in boys at the age of three years [61,62].

Currently, after five years of NBS and follow-up, none of our patients with predicted later-onset forms (mean age at last visit: 3 years) have symptoms or signs of FD. However, further investigations are needed to find the best way for early detection of clinical manifestations in patients with unclassified and later-onset variants. This would allow us to establish an appropriate timing for specific treatments and avoid potential organ damage due to Gb_3_ and lyso-Gb_3_ accumulation. We have provided constant psychological support to the parents from diagnosis and during follow-up, especially to the mothers because this X-linked disease could be a major psychological burden on them [30]. The parents of newborns with later-onset forms were reassured that the newborn would have a normal childhood and that periodic evaluations would help us to determine when therapy is needed in the future.

### 4.4. Biomarkers and Biochemical

Biomarkers and biochemical follow-up show Lyso-Gb_3_ (also known as globotriaosylsphingosine) is the N-deacylated form of Gb_3_ that has been proposed as a specific biomarker for FD [63]. Lyso-Gb_3_ can be measured in plasma or DBS by LC-MS/MS technology, as we have demonstrated [10]. Recently, we studied lyso-Gb_3_ in a large cohort of Fabry patients (*n* = 71) where we observed high levels in males with the classic phenotype and mild-to-moderately elevated levels both in males with the later-onset phenotype and in heterozygous females with the classic phenotype [10]. Storage of Gb_3_ begins in utero [64,65]; therefore, we evaluated the use of this biomarker as a second-tier test by measuring lyso-Gb_3_ in DBS from neonates identified by NBS for FD. We found that only five patients, all with a later-onset pathogenic variant, had abnormal DBS lyso-Gb_3_ at birth, among the 17 tested newborns (of which 12 carrying a pathogenic variant), indicating that a normal result cannot exclude FD and, therefore, cannot be used as a second tier test. At the first visit, plasma lyso-Gb_3_ was abnormal in 11/21 patients, nine of them with a later-onset variant, one carried the p.Ala143Thr variant, and one had the unclassified p.Leu286Val variant. Interestingly, patient #10 had a very low α-GAL A activity (0.73 µmol/L/h) accompanied by the highest level of plasma lyso-Gb_3_ that we found in our neonates (2.98 nmol/L). He carries the p.Arg356Gly variant, considered likely associated with a later-onset phenotype. Moreover, we found an inverse linear correlation between DBS lyso-Gb_3_ /plasma lyso-Gb_3_ and DBS α-GAL A activity at birth/first visit (r: −0.28 and −0.41, respectively). This finding suggests that lower enzyme activity corresponds to a higher storage from birth. Although a normal DBS lyso-Gb_3_ value cannot rule out FD, its use as a specific marker in the diagnostic process is still valid. However, we believe that the use of lyso-Gb_3_ as a second-tier test in newborn screening programs needs further evaluation. 

**Figure 2:**
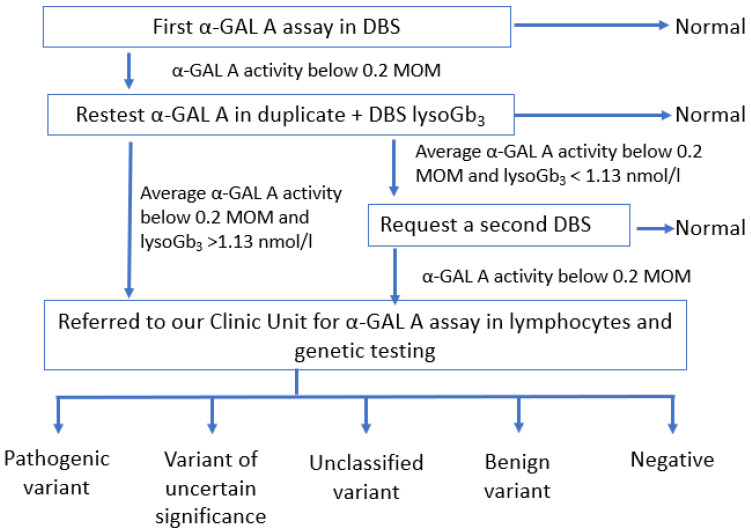
Proposal for a diagnostic algorithm of male newborn screening for Fabry disease. MOM: multiple of median; DBS: dried blood spot.

In light of our five-year-experience with NBS for FD, we propose a new screening algorithm (Figure 2) that is simplified with respect to our previous protocol [28]. In the new algorithm, if the first DBS had a reduced α-GAL A activity and lyso-Gb_3_ levels were above the cut-off, the newborn must be referred directly to a Pediatric Unit without requiring a second DBS. Moreover, biomarkers play an important role during follow-up. A recent case report showed that lyso-Gb_3_ may be elevated in the first days of life and that it increases significantly during infancy in patients affected by a classic (severe) form of FD [66]. In our patients affected by later-onset FD, we found that the levels of plasma lyso-Gb_3_ gradually increased with age, suggesting that there may be a progressive and insidious storage, even in milder forms (Figure 1a). At the last visit (between 0.5 months and 5.5 years of age), plasma lyso-Gb_3_ was above the reference value in 17/19 patients, of which 11 had a later-onset form (all tested), three carried the p.Ala143Thr variant (very mild increase, except in patient #11 who also carried an intronic variant, with a higher although still moderate value of 0.85 nmol/L), and three carried unclassified variants (very slight increase, values in two of these infants were near the upper value of the normal range) (Figure 1a,b). A statistically significant difference was found between plasma lyso-Gb_3_ values of patients carrying a known pathogenic variant (later-onset variant) and patients with unclassified variants or the p.Ala143Thr variant at last visit, but this difference was not statistically significant in the neonatal period. Of note, we found that all patients carrying the p.Asn215Ser variant (patients #4, #17, and #18) had plasma lyso-Gb_3_ levels above the cut-off at birth. These values progressively increased and, interestingly, the highest value was found in the oldest patient (3.91 nmol/L at 4.5 years), suggesting progressive and insidious storage, even in an asymptomatic child. However, all our patients carrying unclassified variants had normal lyso-Gb_3_ at birth and it remained in the normal range in three of four patients at the last visit. Only in case #15, carrying one previously unreported variant in cis with the likely benign p.S126G variant, showed a mild increase in plasma lyso-Gb_3_ (0.92 nmol/L). Nevertheless, we believe that further studies are needed to assess the exact value of lyso-Gb_3_ in neonates carrying unclassified variants. Whether a slight increase in lyso-Gb_3_ has any clinical significance has not been proven and also warrants further studies.

The high incidence of later-onset forms has raised ethical issues regarding the conduct of a NBS program. Detection in the newborn period may have a negative psychological impact on parents and carries the risk that these children, defined as “patient-in-waiting”, are labelled and overmedicated. Moreover, it increases the costs for diagnostic laboratory testing and follow-up visits [67]. However, an early diagnosis of later-onset forms may also have several advantages. A significant number of patients with later-onset forms currently remain mis- or undiagnosed for many years. The implementation of NBS could avoid this “diagnostic odyssey”, allowing timely treatment and subsequently better outcomes [24]. Interestingly, in a recent study, an interview among adult patients with lysosomal diseases was conducted on their opinion toward NBS. The majority of participants agreed with the implementation of NBS, in particular, all patients with FD were in favor of NBS because it may allow for the initiation of earlier treatment and prevent irreversible organ damage [68].

Moreover, their identification allows physicians to perform cascade genotyping in at risk family members and identify undiagnosed relatives [69]. Furthermore, NBS will help to better understand the natural course of the disease.

Our study on NBS for FD shows some limitations. Regarding the role of the biomarker lyso-Gb_3_ as a second-tier test in NBS for FD, our experience confirms, as previously reported [70,71], that it is not reliable and cannot be used to reduce the recall rate. However, lyso-Gb_3_, when elevated in DBS, makes the application of the diagnostic algorithm easier (Figure 2). During follow-up, plasma lyso-Gb_3_ is very useful for the biochemical monitoring of patients.

We did not detect any heterozygotes among the 83,857 newborn females screened, which confirms that the current enzyme-based NBS approach misses most female carriers due to X-chromosome inactivation [72]. This is the major limitation of any enzymatic screening for FD [73]. These findings indicate that NBS for FD is more effective and cost beneficial when it is limited to male newborns. Some authors suggest first-tier screening with *GLA* gene sequencing in female newborns. This method was applied in Taiwan [29,74,75,76] where 21 variants account for approximately 98% of variants [76]. In Italy, mutational heterogeneity hampers the use of molecular analysis for high-throughput screening, which could also increase the number of variants of uncertain significance (VUS) [37]. Moreover, even when a GLA variant is known to be pathogenic, due to the X-chromosome inactivation, females might occasionally remain asymptomatic throughout life [6,77]. However, panels of genes may also be considered in the future, specifically due to improved technologies since they allow for the newborn screening of multiple diseases with the caveats of the difficulties in the interpretation of variants of unknown significance.

## 5. Conclusions

Our study confirms that NBS for Fabry disease is feasible through the measurement of α-GAL A enzyme activity in DBS and should be evaluated for inclusion in the national NBS program. Biomarkers like lyso-Gb_3_ are useful in the NBS protocol for diagnosis and follow-up. In accordance with other NBS studies, FD appears to be more frequent than previously estimated clinically, therefore NBS may help to improve the diagnosis of many unrecognized patients. However, several issues still need further study: (1) the significance of mildly elevated plasma lyso-Gb_3_; (2) the absence of a reliable second-tier test to reduce the recall rate; (3) poor detection of heterozygous females; (4) the clinical interpretation of unclassified and uncertain genetic variants; and (5) the impact of early diagnosis on patients with later-onset forms. Our overall experience in NBS for FD is positive, and the project is moving forward with the aim of gaining a better understanding of the disease and better care for the patients. 

## Figures and Tables

**Table 1:** Summary of the methods and results from pilot and regular screening programs for Fabry disease worldwide.

Publication Year	Study Period	Region	Method	Number of NBS Samples	Positive NBS/ Patients Referred to Clinic	Confirmed Patients	Confirmed Male Patients	Reported Incidence *
2006	2003–2005	Italy [33]	Fluorometric enzyme assay	37,104 (only males)	12 (m)	12	12	1:3,100 (m)
2009	2006–2008	Taiwan [34]	Fluorometric enzyme assay	171,977 (m 90,288)	94 (m 91)	75	73	1:3821 (m 1:1237)
2017	2008	Spain [27]	Fluorometric enzyme assay	14,600 (m 7575)	106 (m 68)	37	20	1:394 (m 1:378) **
2009	2008–2009	Taiwan [35]	Fluorometric enzyme assay	110,027 (m 57,451)	67 (m 58)	45	42	1:2445 (m 1:1368)
2013	2007–2010	Japan [30]	Fluorometric enzyme assay	21,170 (m 10,827)	7 (m 5)	6	5	1:3,024 (m 1:2166)
2012	2010	Austria [26]	MS/MS	34,736 (deidentified)	28	9	6	1:3860
2012	2010–2011	Illinois [36]	Digital microfluidics	8012	11	7	6	1:1145
2012	2011	Hungary [25]	MS/MS	40,024	34	14	6	1:2858
2012	2010–2012	Italy [37]	Fluorometric enzyme assay	3403 (m 1702)	0	0	0	0
2014	2010–2013	Taiwan [38]	MS/MS	191,767	79	64	61	1:2996
2020	2011–2013	California [39]	MS/MS, immunocapture assay, digital microfluidics (comparative)	89,508 (de-identified) (m 44,664)	Variable based on method	50	46	1:1790(m 1:970)
2013	2013	Washington State [22]	MS/MS	108,905 (deidentified) (m 54,800)	16 (m 13)	7	7	1:15558 (m 1:7800)
2015	2013	Missouri [23]	Digital microfluidics	43,701	28	15	15	1:2913
2017	2007–2014	Japan [40]	Fluorometric enzyme assay	2443	2	2	2	1:1222
2018	2008–2014	Taiwan [41]	Fluorometric enzyme assay, then MS/MS	792,247 (m 412,299)	764 (m 425)	324	272	1:2445 (m 1:1515)
2016	2008–2015	Taiwan [42]	Fluorometric enzyme assay, then MS/MS	916,383 (m 476,909)	936 (m 505)	441	324	1:2078 (m 1:1472)
2017	2012–2016	Petroleos Mexicanos Health Services [43]	MS/MS	20,018 (m 10,241)	5	5	5	1:4003 (m 1:2048)
2017	2014–2016	Illinois [24]	MS/MS	219,793	107	32	32	1:6968
2016	2016	Washington [44]	MS/MS	43,000 (deidentified)	8	5	NA	1:8600
2018	2015–2017	Italy [28]	MS/MS	44,411	5	5	5	1:8882
2018	2017	Brazil [45]	Digital microfluidics	10,527	0	0	0	0
2020	2006–2018	Japan [46]	Fluorometric enzyme assay	599,711	138	108	64	1:5552
2019	2013–2019	New York [47]	MS/MS	65,605	31	7	7	1:9372
2020	2018–2019	Taiwan [48]	MS/MS	73,743	4	4	NA	1:18,436

m: males (if indicated); MS/MS: tandem mass spectrometry; NBS: newborn screening; * incidence as reported in the respective studies. It is difficult to make comparison among studies, especially because changes in the classification of variants over time, so that some previously pathogenic variants have been reclassified (e.g., p.Arg118Cys, p.Asp313Tyr and the debated variant p.Ala143Thr). Furthermore, regarding some unclassified variants, it is difficult to predict their pathogenicity (see text); ** only 1 known pathogenic variant, 11 variants of uncertain significance (VUS), 25 polymorphisms (see text).

**Table 2:** Results of the newborn screening program for Fabry disease in northeast Italy (September 2015 to March 2021).

	Males	Females	Total
Screened newborns	89,485	83,857	173,342
Newborns with decreased enzyme activity in the 1st DBS, after retesting in duplicate	44	9	53
Recall %	0.05%	0.01%	0.03%
Newborns with decreased enzyme activity in the 2nd DBS and referred to Clinic Unit for confirmatory testing	22	1	23
Newborns confirmed by low enzyme activity in lymphocytes and *GLA* gene mutation	22	0	22
Pathogenic classical variants	0	0	0
Pathogenic later-onset variants	13	0	13
Benign variants	1	0	1
False-positive results	0	1	1
Unclassified variants	4	0	4
p.Ala143Thr variant	4	0	4
Overall incidence	1:4068	0	1:7879
Pathogenic variants incidence	1:6883	0	1:13,334

Cut-off <0.2 MOM (multiple of median).

**Table 3:** Clinical, biochemical, and molecular results of the patients detected by newborn screening for Fabry disease: baseline and follow-up.

Case	Year of Birth	Gender	Ethnic Origin	DBS AGAL Activity *	DBS LysoGb_3_ (nv < 1.13 nmol/L)	Lymphocytes AGAL Activity **	Plasma LysoGb_3_ at First Visit (nv < 0.43 nmol/L)	cDNA Variation (Protein Variation)	Classification International Fabry Disease Genotype-Phenotype Database [49] ***	Age at Last Visit	Clinical Manifestations	Plasma LysoGb_3_ at the Last Visit (nv < 0.43 nmol/L)
1	2015	M	Europe	3.21	NA	100	**0.53**	c.427G>A (p.Ala143Thr)	Benign	5.5 years	No	**0.54**
2	2015	M	Europe	2.76	NA	9	0.12	c.427G>A (p.Ala143Thr)	Benign	4.5 years	No	**0.48**
3	2015	M	Europe	2.93	NA	354	0.31	c.427G>A (p.Ala143Thr)	Benign	4.5 years	No	0.35
4	2016	M	Europe	0.64	NA	0	**1.07**	c.644 A>G (p.Asn215Ser) + IVS2-77_81del5; IVS4-16A>G; IVS6-22C>T	Later-onset + NA ****	4.5 years	No	**3.91**
5	2016	M	Europe	2.25	1.02	355	0.19	-10C>T; IVS2-77_81del5; IVS4-16A>G; IVS6-22C>T	NA ****	Lost to follow-up		
6	2016	M	Europe	3.45	NA	346	0.27	c.737C>T (p.Thr246Ile)	NA	4.5 years	No	**0.43**
7	2016	M	East Asia	0.77	0.79	143	0.3	IVS4 + 919G>A	Later-onset	4 years	No	**1.12**
8	2016	M	North Africa	0.72	**1.79**	66	**1.02**	c.1088G>A (p.Arg363His)	Later-onset	4 years	No	**1.91**
9	2016	M	East Asia	1.16	0.62	222	**0.54**	IVS4 + 919G>A	Later-onset	4 years	No	**1.7**
10	2016	M	Europe	0.73	**2.17**	27	**2.98**	c.1066 C>G (p.Arg356Gly)	Likely later-onset	4 years	No	**3.71**
11	2017	M	Europe	2.05	0.54	316	0.36	c.427G>A (p.Ala143Thr) + IVS4-61_60delGT	Benign + NA	3.5 years	No	**0.85**
12	2017	M	Europe	1.37	**1.25**	NA	**0.85**	c.153G>A (p.Met51Ile)	Later-onset	3.5 years	No	**0.82**
13	2017	M	West Africa	1.51	0.96	0.73	0.26	c.1067G>A (p.Arg356Gln)	Later-onset	3 years	No	**0.43**
14	2018	M	Europe	0.79	0.41	NA	0.2	c.868A>C (p.Met290Leu) + -10C>T; IVS2-77_81del5; IVS4-16A>G; IVS6-22C>T	Later-onset + NA ****	Lost to follow-up		
15	2018	M	Europe	0.87	0.73	0.82	0.35	c.347G>C (p.Gly116Ala) + c.376A>G (p.Ser126Gly) + -10C>T; IVS2-77_81del5; IVS4-16A>G; IVS6-22C>T	NA + likely benign + NA ****	2.5 years	No	**0.92**
16	2018	M	Europe	1.28	0.22	3.44	**0.49**	c.856C>G (p.Leu286Val)	NA	2 years	No	**0.43**
17	2019	M	Europe	0.63	0.5	1.84	**0.82**	c.644A>G (p.Asn215Ser)	Later-onset	1 year	No	**1.95**
18	2019	M	Europe	1.4	**1.1**	3.35	**1.32**	c.644A>G (p.Asn215Ser)	Later-onset	1.5 years	No	NA
19	2019	M	North Africa	0.77	**2.7**	2.41	**1**	c.1088G>A (p.Arg363His)	Later-onset	1.5 years	No	**1.49**
20	2019	M	West Africa	1.63	1.07	1.05	NA	c.1067G>A (p.Arg356Gln)	Later-onset	1 year	No	**0.56**
21	2020	M	Europe	1.12	1.07	2.18	0.41	c.856C>G (p.Leu286Val)	NA	10 d	No	0.41
22	2020	M	Europe	1.88	0.77	1.94	0.44	c.868A>C (p.Met290Leu)	Later-onset	6 m	No	**0.51**

AGAL: α-GAL A; DBS: dried blood spot; N/A: not available; nv: normal values; M: males; the pathological values are marked in bold. * First cut-off used until May 2016 (case number 6) 3.76 µmol/L/h, after 9 months of screening it was reset to 2.3 µmol/L/h; ** until June 2017 (case number 11) a fluorometric method was used (nv 360-1374 mU/L), then we used a MS/MS technology (in neonate nv < 4.38 nmol/h/mg protein); *** last accessed on 24 May 2021; **** IVS4-16A > G, IVS6-22C > T, -10C > T are classified as benign variants in other databases (e.g., Fabry-Gen-Phen [51]).

## Supplementary Materials

The following are available online at https://www.mdpi.com/article/10.3390/biom11070951/s1, Table S1: Follow up of patients detected by newborn screening for Fabry disease.

## References

[1] Germain D.P. (2010). Fabry disease. Orphan J. Rare Dis..

[2] Kok K., Zwiers K.C., Boot R.G., Overkleeft H.S. (2021). Fabry Disease: Molecular Basis, Pathophysiology, Diagnostics and Potential Therapeutic Directions. Biomolecules.

[3] Burlina A.P., Politei J., Burlina A.P. (2018). Fabry disease. Neurometabolic Hereditary Diseases of Adults.

[4] Desnick R.J., Ioannou Y.A., Eng C.M. (2021). A-Galactosidase A Deficiency: Fabry Disease.

[5] Germain D.P., Brand E., Burlina A., Cecchi F., Garman S.C., Kempf J., Laney D.A., Linhart A., Maródi L., Nicholls K. (2018). Phenotypic Characteristics of the p.Asn215Ser (p.N215S) *GAL* Mutation in Male and Female Patients with Fabry Disease: A Multicenter Fabry Registry Study. Mol. Genet. Genom. Med..

[6] Echevarria L., Benistan K., Toussaint A., Dubourg O., Hagege A.A., Eladari D., Jabbour F., Beldjord C., De Mazancourt P., Germain D.P. (2016). X-chromosome inactivation in female patients with Fabry disease. Clin. Genet..

[7] Massaccesi L., Burlina A., Baquero C.J., Goi G., Burlina A.P., Tettamanti G. (2011). Whole-Blood Alpha-D-Galactosidase A Activity for the Identification of Fabry’s Patients. Clin. Biochem..

[8] Gal A., Beck M., Höppner W., Germain D.P. (2017). Clinical utility gene card for Fabry disease—Update 2016. Eur. J. Hum. Genet..

[9] Rombach S.M., Dekker N., Bouwman M.G., Linthorst G.E., Zwinderman A.H., Wijburg F.A., Kuiper S., vd Bergh Weerman M.A., Groener J.E.M., Poorthuis B.J. (2010). Plasma Globotriaosylsphingosine: Diagnostic Value and Relation to Clinical Manifestations of Fabry Disease. Biochim. Biophys. Acta (BBA) Mol. Basis Dis..

[10] Polo G., Burlina A.P., Ranieri E., Colucci F., Rubert L., Pascarella A., Duro G., Tummolo A., Padoan A., Plebani M. (2019). Plasma and Dried Blood Spot Lysosphingolipids for the Diagnosis of Different Sphingolipidoses: A Comparative Study. Clin. Chem. Lab. Med..

[11] Effraimidis G., Feldt-Rasmussen U., Rasmussen Å.K., Lavoie P., Abaoui M., Boutin M., Auray-Blais C. (2020). Globotriaosylsphingosine (Lyso-Gb_3_) and Analogues in Plasma and Urine of Patients with Fabry Disease and Correlations with Long-Term Treatment and Genotypes in a Nationwide Female Danish Cohort. J. Med. Genet..

[12] Schiffmann R., Murray G.J., Treco D., Daniel P., Sellos-Moura M., Myers M., Quirk J.M., Zirzow G.C., Borowski M., Loveday K. (2000). Infusion of alpha-Galactosidase A Reduces Tissue Globotriaosylceramide Storage in Patients with Fabry Disease. Proc. Natl. Acad. Sci. USA.

[13] Eng C.M., Guffon N., Wilcox W.R., Germain D.P., Lee P., Waldek S., Caplan L., Linthorst G.E., Desnick R.J. (2001). For The International Collaborative Fabry Disease Study Group: Safety and efficacy of recombinant human alphagalactosidase A—replacement therapy in Fabry’s disease. N. Engl. J. Med..

[14] Germain D.P., Arad M., Burlina A., Elliott P.M., Falissard B., Feldt-Rasmussen U., Hilz M.J., Hughes D.A., Ortiz A., Wanner C. (2018). The effect of enzyme replacement therapy on clinical outcomes in female patients with Fabry disease—A systematic literature review by a European panel of experts. Mol. Genet. Metab..

[15] Ortiz A., Germain D.P., Desnick R.J., Politei J., Mauer M., Burlina A., Eng C., Hopkin R.J., Laney D., Linhart A. (2018). Fabry Disease Revisited: Management and Treatment Recommendations for Adult Patients. Mol. Genet. Metab..

[16] Germain D.P., Charrow J., Desnick R.J., Guffon N., Kempf J., Lachmann R.H., Lemay R., Linthorst G.E., Packman S., Scott C.R. (2015). Ten-Year Outcome of Enzyme Replacement Therapy with Agalsidase Beta in Patients with Fabry Disease. J. Med. Genet..

[17] Chamoles N.A., Blanco M., Gaggioli D. (2001). Fabry disease enzymatic diagnosis in dried blood spot on filter paper. Clinica Chimica Acta.

[18] Gelb M.H., Turecek F., Scott C.R., Chamoles N.A. (2006). Direct Multiplex Assay of Enzymes in Dried Blood Spots by Tandem Mass Spectrometry for the Newborn Screening of Lysosomal Storage Disorders. J. Inherit. Metab Dis..

[19] Zhang X.K., Elbin C.S., Chuang W.-L., Cooper S.K., Marashio C.A., Beauregard C., Keutzer J.M. (2008). Multiplex Enzyme Assay Screening of Dried Blood Spots for Lysosomal Storage Disorders by Using Tandem Mass Spectrometry. Clin. Chem..

[20] Sista R.S., Eckhardt A.E., Wang T., Graham C., Rouse J.L., Norton S.M., Srinivasan V., Pollack M.G., Tolun A.A., Bali D. (2011). Digital Microfluidic Platform for Multiplexing Enzyme Assays: Implications for Lysosomal Storage Disease Screening in Newborns. Clin. Chem..

[21] Mechtler T.P., Metz T.F., Müller H.G., Ostermann K., Ratschmann R., De Jesus V.R., Shushan B., Di Bussolo J.M., Herman J.L., Herkner K.R. (2012). Short-Incubation Mass Spectrometry Assay for Lysosomal Storage Disorders in Newborn and High-Risk Population Screening. J. Chromatogr. B.

[22] Scott C.R., Elliott S., Buroker N., Thomas L.I., Keutzer J., Glass M., Gelb M.H., Turecek F. (2013). Identification of Infants at Risk for Developing Fabry, Pompe or Mucopolysaccharidosis-I from Newborn Blood Spots by Tandem Mass Spectrometry. J. Pediatr..

[23] Hopkins P.V., Campbell C., Klug T., Rogers S., Raburn-Miller J., Kiesling J. (2015). Lysosomal Storage Disorder Screening Implementation: Findings from the First Six Months of Full Population Pilot Testing in Missouri. J. Pediatr..

[24] Burton B.K. (2017). Newborn Screening for Lysosomal Storage Disorders in Illinois: The Initial 15-Month Experience. J. Pediatr..

[25] Wittmann J., Karg E., Turi S., Legnini E., Wittmann G., Giese A.-K., Lukas J., Gölnitz U., Klingenhäger M., Bodamer O., SSIEM (2012). Newborn Screening for Lysosomal Storage Disorders in Hungary. JIMD Reports—Case and Research Reports, 2012/3.

[26] Mechtler T.P. (2012). Neonatal Screening for Lysosomal Storage Disorders: Feasibility and Incidence from a Nationwide Study in Austria. Lancet.

[27] Colon C., Ortolano S., Melcon-Crespo C., Alvarez J.V., Lopez-Suarez O.E., Couce M.L., Fernández-Lorenzo J.R. (2017). Newborn Screening for Fabry Disease in the North-West of Spain. Eur. J. Pediatr..

[28] Burlina A.B., Polo G., Salviati L., Duro G., Zizzo C., Dardis A., Bembi B., Cazzorla C., Rubert L., Zordan R. (2018). Newborn Screening for Lysosomal Storage Disorders by Tandem Mass Spectrometry in North East Italy. J. Inherit. Metab Dis..

[29] Chien Y.-H., Lee N.-C., Chiang S.-C., Desnick R.J., Hwu W.-L. (2012). Fabry Disease: Incidence of the Common Later-Onset α-Galactosidase A IVS4+919G→A Mutation in Taiwanese Newborns—Superiority of DNA-Based to Enzyme-Based Newborn Screening for Common Mutations. Mol. Med..

[30] Inoue T., Hattori K., Ihara K., Ishii A., Nakamura K., Hirose S. (2013). Newborn Screening for Fabry Disease in Japan: Prevalence and Genotypes of Fabry Disease in a Pilot Study. J. Hum. Genet..

[31] Meikle P.J. (1999). Prevalence of Lysosomal Storage Disorders. JAMA.

[32] Richards S., Aziz N., Bale S., Bick D., Das S., Gastier-Foster J., Grody W.W., Hedge M., Lyon E., Spector E. (2015). Standards and guidelines for the interpretation of sequence variants: A joint consensus recommendation of the American College of Medical Genetics and Genomics and the Association for Molecular Pathology. Genet. Med..

[33] Spada M., Pagliardini S., Yasuda M., Tukel T., Thiagarajan G., Sakuraba H., Ponzone A., Desnick R.J. (2006). High Incidence of Later-Onset Fabry Disease Revealed by Newborn Screening. Am. J. Hum. Genet..

[34] Hwu W.-L., Chien Y.-H., Lee N.-C., Chiang S.-C., Dobrovolny R., Huang A.-C., Yeh H.-Y., Chao M.-C., Lin S.-J., Kitagawa T. (2009). Newborn Screening for Fabry Disease in Taiwan Reveals a High Incidence of the Later-Onset *GLA* Mutation c.936+919G>A (IVS4+919G>A). Hum. Mutat..

[35] Lin H.-Y., Chong K.-W., Hsu J.-H., Yu H.-C., Shih C.-C., Huang C.-H., Lin S.-J., Chen C.-H., Chiang C.-C., Ho H.-J. (2009). High Incidence of the Cardiac Variant of Fabry Disease Revealed by Newborn Screening in the Taiwan Chinese Population. Circ. Cardiovasc. Genet..

[36] Burton B., Charrow J., Angle B., Widera S., Waggoner D. (2012). A pilot newborn screening program for lysosomal storage disease in Illinois. Mol. Genet. Metab..

[37] Paciotti S., Persichetti E., Pagliardini S., Deganuto M., Rosano C., Balducci C., Codini M., Filocamo M., Menghini A.R., Pagliardini V. (2012). First Pilot Newborn Screening for Four Lysosomal Storage Diseases in an Italian Region: Identification and Analysis of a Putative Causative Mutation in the GBA Gene. Clin. Chim. Acta.

[38] Liao H.-C., Chiang C.-C., Niu D.-M., Wang C.-H., Kao S.-M., Tsai F.-J., Huang Y.-H., Liu H.-C., Huang C.-K., Gao H.-J. (2014). Detecting Multiple Lysosomal Storage Diseases by Tandem Mass Spectrometry—A National Newborn Screening Program in Taiwan. Clin. Chim. Acta.

[39] Sanders K.A., Gavrilov D.K., Oglesbee D., Raymond K.M., Tortorelli S., Hopwood J.J., Lorey F., Majumdar R., Kroll C.A., McDonald A.M. (2020). A Comparative Effectiveness Study of Newborn Screening Methods for Four Lysosomal Storage Disorders. IJNS.

[40] Chinen Y., Nakamura S., Yoshida T., Maruyama H., Nakamura K. (2017). A New Mutation Found in Newborn Screening for Fabry Disease Evaluated by Plasma Globotriaosylsphingosine Levels. Hum. Genome Var..

[41] Liao H.-C., Hsu T.-R., Young L., Chiang C.-C., Huang C.-K., Liu H.-C., Niu D.-M., Chen Y.-J. (2018). Functional and Biological Studies of α-Galactosidase A Variants with Uncertain Significance from Newborn Screening in Taiwan. Mol. Genet Metab..

[42] Hsu T.-R. (2016). Later Onset Fabry Disease, Cardiac Damage Progress in Silence. J. Am. Coll. Cardiol..

[43] Navarrete-Martínez J.I., Limón-Rojas A.E., Gaytán-García M.d.J., Reyna-Figueroa J., Wakida-Kusunoki G., Delgado-Calvillo M.d.R., Cantú-Reyna C., Cruz-Camino H., Cervantes-Barragán D.E. (2017). Newborn Screening for Six Lysosomal Storage Disorders in a Cohort of Mexican Patients: Three-Year Findings from a Screening Program in a Closed Mexican Health System. Mol. Genet. Metab..

[44] Elliott S., Buroker N., Cournoyer J.J., Potier A.M., Trometer J.D., Elbin C., Schermer M.J., Kantola J., Boyce A., Turecek F. (2016). Pilot Study of Newborn Screening for Six Lysosomal Storage Diseases Using Tandem Mass Spectrometry. Mol. Genet. Metab..

[45] Camargo Neto E., Schulte J., Pereira J., Bravo H., Sampaio-Filho C., Giugliani R. (2018). Neonatal Screening for Four Lysosomal Storage Diseases with a Digital Microfluidics Platform: Initial Results in Brazil. Genet. Mol. Biol..

[46] Sawada T., Kido J., Yoshida S., Sugawara K., Momosaki K., Inoue T., Tajima G., Sawada H., Mastumoto S., Endo F. (2020). Newborn Screening for Fabry Disease in the Western Region of Japan. Mol. Genet. Metab. Rep..

[47] Wasserstein M.P., Caggana M., Bailey S.M., Desnick R.J., Edelmann L., Estrella L., Holzman I., Kelly N.R., Kornreich R., Kupchik S.G. (2019). The New York Pilot Newborn Screening Program for Lysosomal Storage Diseases: Report of the First 65,000 Infants. Genet. Med..

[48] Chien Y.-H. (2020). Newborn Screening for Morquio Disease and Other Lysosomal Storage Diseases: Results from the 8-Plex Assay for 70,000 Newborns. Orphanet J. Rare Dis..

[49] International Fabry Disease Genotype-Phenotype Database. www.dbfgp.org.

[50] Germain D.P., Oliveira J.P., Bichet D.G., Yoo H.-W., Hopkin R.J., Lemay R., Politei J., Wanner C., Wilcox W.R., Warnock D.G. (2020). Use of a Rare Disease Registry for Establishing Phenotypic Classification of Previously Unassigned *GLA* Variants: A Consensus Classification System by a Multispecialty Fabry Disease Genotype–Phenotype Workgroup. J. Med. Genet..

[51] Fabry-Gen-Phen: The Fabry Working Group Genotype Phenotype Database. http://fabrygenphen.com/.

[52] Lavalle L., Thomas A.S., Beaton B., Ebrahim H., Reed M., Ramaswami U., Elliott P., Mehta A.B., Hughes D.A. (2018). Phenotype and Biochemical Heterogeneity in Late Onset Fabry Disease Defined by N215S Mutation. PLoS ONE.

[53] Lenders M., Weidemann F., Kurschat C., Canaan-Kühl S., Duning T., Stypmann J., Schmitz B., Reiermann S., Krämer J., Blaschke D. (2016). Alpha-Galactosidase A p.A143T, a Non-Fabry Disease-Causing Variant. Orphanet J. Rare Dis..

[54] Koca S., Tümer L., Okur İ., Erten Y., Bakkaloğlu S., Biberoğlu G., Kasapkara Ç., Küçükçongar A., Dalgıç B., Oktar S.Ö. (2019). High Incidence of Co-Existing Factors Significantly Modifying the Phenotype in Patients with Fabry Disease. Gene.

[55] Elliott P., Baker R., Pasquale F., Quarta G., Ebrahim H., Mehta A.B., Hughes D.A., On Behalf of the ACES Study Group (2011). Prevalence of Anderson-Fabry Disease in Patients with Hypertrophic Cardiomyopathy: The European Anderson-Fabry Disease Survey. Heart.

[56] Brabander I.D. (2013). Phenotypical Characterization of α-Galactosidase A Gene Mutations Identified in a Large Fabry Disease Screening Program in Stroke in the Young. Clin. Neurol. Neurosurg..

[57] Terryn W., Vanholder R., Hemelsoet D., Leroy B.P., Van Biesen W., De Schoenmakere G., Wuyts B., Claes K., De Backer J., De Paepe G., Zschocke J., Gibson K.M., Brown G., Morava E., Peters V. (2012). Questioning the Pathogenic Role of the GLA p.Ala143Thr “Mutation” in Fabry Disease: Implications for Screening Studies and ERT. JIMD Reports—Case and Research Reports, 2012/5.

[58] Krüger R., Tholey A., Jakoby T., Vogelsberger R., Mönnikes R., Rossmann H., Beck M., Lackner K.J. (2012). Quantification of the Fabry Marker LysoGb3 in Human Plasma by Tandem Mass Spectrometry. J. Chromatogr. B.

[59] The Genome Aggregation Database. https://gnomad.broadinstitute.org.

[60] Oqvist B., Brenner B.M., Oliveira J.P., Ortiz A., Schaefer R., Svarstad E., Wanner C., Zhang K., Warnock D.G. (2009). Nephropathy in Fabry Disease: The Importance of Early Diagnosis and Testing in High-Risk Populations. Nephrol. Dial. Transplant..

[61] Ries M., Ramaswami U., Parini R., Lindblad B., Whybra C., Willers I., Gal A., Beck M. (2003). The Early Clinical Phenotype of Fabry Disease: A Study on 35 European Children and Adolescents. Eur. J. Pediatr..

[62] Germain D.P., Fouilhoux A., Decramer S., Tardieu M., Pillet P., Fila M., Rivera S., Deschênes G., Lacombe D. (2019). Consensus recommendations for diagnosis, management and treatment of Fabry disease in paediatric patients. Clin. Genet..

[63] Aerts J.M., Groener J.E., Kuiper S., Donker-Koopman W.E., Strijland A., Ottenhoff R., van Roomen C., Mirzaian M., Wijburg F.A., Linthorst G.E. (2008). Elevated Globotriaosylsphingosine Is a Hallmark of Fabry Disease. Proc. Natl. Acad. Sci. USA.

[64] Vedder A.C., Strijland A., Weerman M.A.v.B., Florquin S., Aerts J.M.F.G., Hollak C.E.M. (2006). Manifestations of Fabry Disease in Placental Tissue. J. Inherit. Metab. Dis..

[65] Desnick R.J. (2007). Prenatal Diagnosis of Fabry Disease. Prenat. Diagn..

[66] Spada M., Kasper D., Pagliardini V., Biamino E., Giachero S., Porta F. (2017). Metabolic Progression to Clinical Phenotype in Classic Fabry Disease. Ital. J. Pediatr..

[67] Timmermans S., Buchbinder M. (2010). Patients-in-waiting: Living between sickness and health in the genomics era. J. Health Soc. Behav. J. Health Soc. Behav..

[68] Lisi E., Ali N. (2021). Opinions of adults affected with later-onset lysosomal storage diseases regarding newborn screening: A qualitative study. J. Genet. Couns..

[69] Germain D.P., Moiseev S., Suárez-Obando F., Al Ismaili F., Al Khawaja H., Altarescu G., Barreto F.C., Haddoum F., Hadipour F., Maksimova I. (2021). The benefits and challenges of family genetic testing in rare genetic diseases-lessons from Fabry disease. Mol. Genet. Genomic Med..

[70] Burlina A.B., Polo G., Rubert L., Gueraldi D., Cazzorla C., Duro G., Salviati L., Burlina A.P. (2019). Implementation of Second-Tier Tests in Newborn Screening for Lysosomal Disorders in North Eastern Italy. IJNS.

[71] Johnson B., Mascher H., Mascher D., Legnini E., Hung C.Y., Dajnoki A., Chien Y.-H., Maródi L., Hwu W.-L., Bodamer O.A. (2013). Analysis of Lyso-Globotriaosylsphingosine in Dried Blood Spots. Ann. Lab. Med..

[72] Hsu T.-R., Niu D.-M. (2018). Fabry Disease: Review and Experience during Newborn Screening. Trends Cardiovasc. Med..

[73] Caudron E., Prognon P., Germain D.P. (2015). Enzymatic diagnosis of Fabry disease using a fluorometric assay on dried blood spots: An alternative methodology. Eur. J. Med. Genet..

[74] Tai C.-L., Liu M.-Y., Yu H.-C., Chiang C.-C., Chiang H., Suen J.-H., Kao S.-M., Huang Y.-H., Wu T.J.-T., Yang C.-F. (2012). The Use of High Resolution Melting Analysis to Detect Fabry Mutations in Heterozygous Females via Dry Bloodspots. Clin. Chim. Acta.

[75] Lee S.-H., Li C.-F., Lin H.-Y., Lin C.-H., Liu H.-C., Tsai S.-F., Niu D.-M. (2014). High-Throughput Detection of Common Sequence Variations of Fabry Disease in Taiwan Using DNA Mass Spectrometry. Mol. Genet. Metab..

[76] Lu Y.-H., Huang P.-H., Wang L.-Y., Hsu T.-R., Li H.-Y., Lee P.-C., Hsieh Y.-P., Hung S.-C., Wang Y.-C., Chang S.-K. (2018). Improvement in the Sensitivity of Newborn Screening for Fabry Disease among Females through the Use of a High-Throughput and Cost-Effective Method, DNA Mass Spectrometry. J. Hum. Genet..

[77] MacDermot K.D. (2001). Anderson-Fabry Disease: Clinical Manifestations and Impact of Disease in a Cohort of 60 Obligate Carrier Females. J. Med. Genet..

