# Supplementary material for: Newborn Screening for Fabry Disease in Northeastern Italy: Results of Five Years of Experience
Source: Biomolecules. 2021 Jun 27;11(7):951. doi: 10.3390/biom11070951 (PMC8301924; doi:10.3390/biom11070951)
Supplement: Supplementary file 1 [file biomolecules-11-00951-s001.zip › biomolecules-1253797-supplementary.pdf]

Table S1: Follow up of patients detected by newborn screening for Fabry disease

Case	Age at last visit	Clinical evaluation (angiokeratomas, hypohidrosis, gastrointestinal symptoms, limb pain)	Heart examination	Electrocardiogram	eGFR (Schwartz formula)	Uprot/Ucrea (mg/mmol)	Microalbuminuria (mg/g creatinine)
1	5.5 yrs	Normal	Normal	Normal	222.94	18.75	7.1
2	4.5 yrs	Normal	Normal	Normal	136.86	20.62	2.6
3	4.5 yrs	Normal	Normal	Normal	158.12	16.12	20
4	4.5 yrs	Normal	Normal	Normal	162.17	17.34	4.6
5	LOST TO FOLLOW-UP						
6	4.5 yrs	Normal	Normal	Normal	166.52	8.94	11.6
7	4 yrs	Normal	Normal	Normal	123.45	13.95	3.6
8	4 yrs	Normal	Normal	Normal	163.28	NA	NA
9	4 yrs	Normal	Normal	Normal	166.66	23.67	9.6
10	4 yrs	Normal	Normal	Normal	140.25	10.04	20.12
11	3.5 yrs	Normal	Normal	Normal	148.08	19.94	13.4
12	3.5 yrs	Normal	Normal	Normal	128.75	18.51	5.3
13	3 yrs	Normal	Normal	Normal	184.64	NA	NA
14	LOST TO FOLLOW-UP						
15	2.5 yrs	Normal	Normal	Normal	128.56	31	13.7
16	2 yrs	Normal	Normal	Normal	184.8	58.13	40.1

17	1 yr	Normal	Normal	Normal	130	44.64	36.4
18	1.5 yrs	Normal	Normal	Normal	168.67	22.47	31.1
19	1.5 yrs	Normal	Normal	Normal	157.14	NA	NA
20	1 yr	Normal	Normal	Normal	185	NA	NA
21	10 days	Normal	Normal	Normal	71.71	7.19	20.8
22	6 m	Normal	Normal	Normal	110	26.74	16.5

Abbreviations: yrs: years; m: month; eGFR: estimated glomerular filtration rate; Uprot/Ucrea: U protein/U creatinine; NA: not available.
